# Supplementary material for: Evolution of an assembly factor-based subunit contributed to a novel NDH-PSI supercomplex formation in chloroplasts
Source: Nat Commun. 2021 Jun 17;12:3685. doi: 10.1038/s41467-021-24065-0 (PMC8211685; doi:10.1038/s41467-021-24065-0)
Supplement: Supplementary file 1 — Supplementary Figures. [file 41467_2021_24065_MOESM1_ESM.pdf]

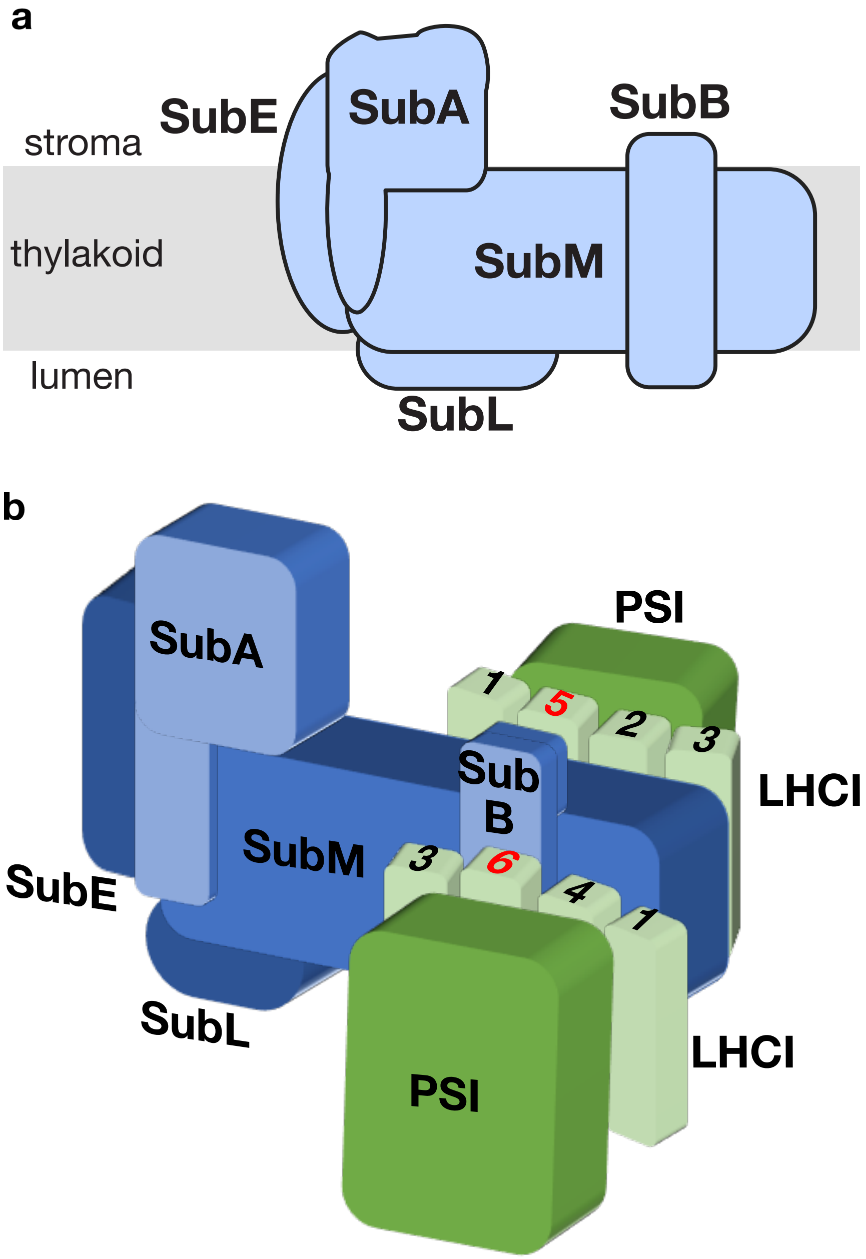

Supplementary Figure 1. Structural model of the NDH-PSI supercomplex

(a) Five subcomplexes of the NDH complex are shown. (b) The NDH complex, LHCI, and PSI are colored blue, pale green, and green, respectively. Lhca1 - Lhca6 are indicated by their numbers in LHCI.

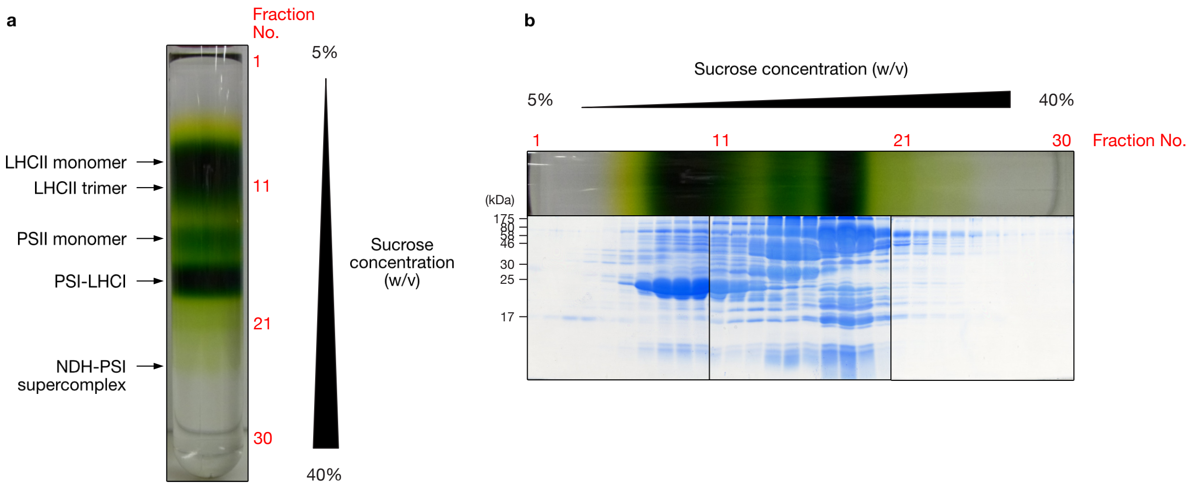

Supplementary Figure 2. Separation of photosynthetic electron transport complexes by SDG ultracentrifugation

(a) Protein complexes of the chloroplast membrane of WT were solubilized and separated by SDG ultracentrifugation. LHCII (monomer and trimer), PSII monomer, and PSI-LHCI were identified based on previous studies[1,2]. (b) Fractions of WT SDG were subjected to SDS-PAGE and CBB staining. The photos of the SDG tube and the SDS-PAGE lanes were aligned according to the major green bands (LHCII, PSII monomer, and PSI-LHCI) and the pattern of the CBB-stained gels. Each experiment was performed in at least twice.

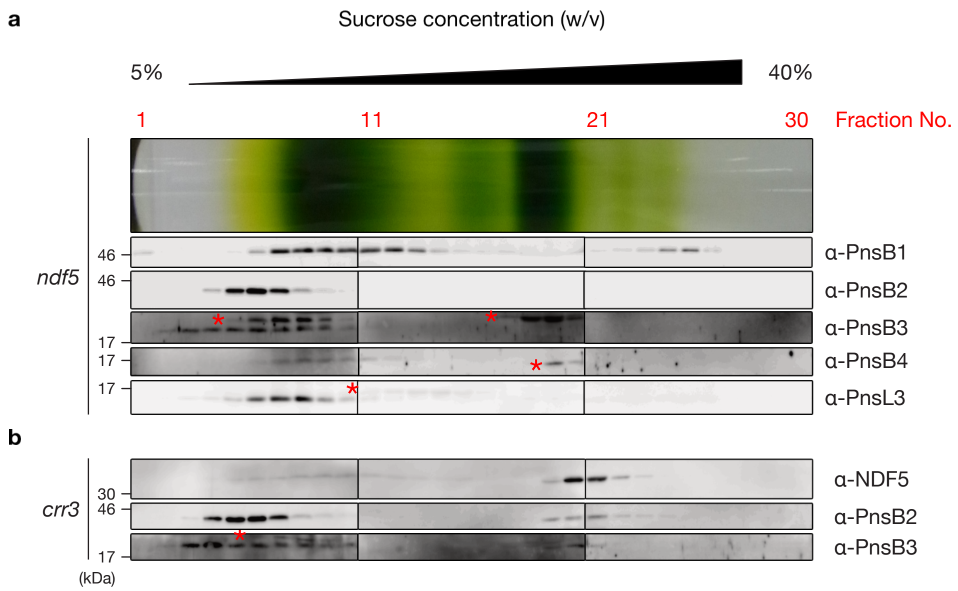

Supplementary Figure 3. NDF5 was required for the early stage of SubB assembly. Protein complexes in the chloroplast membrane isolated from (a) *ndf5* and (b) *crr3* mutants were separated by SDG ultracentrifugation. SubB subunits and NDF5 were detected by immunoblotting, as in Figure 2. Each experiment was performed in at least twice.

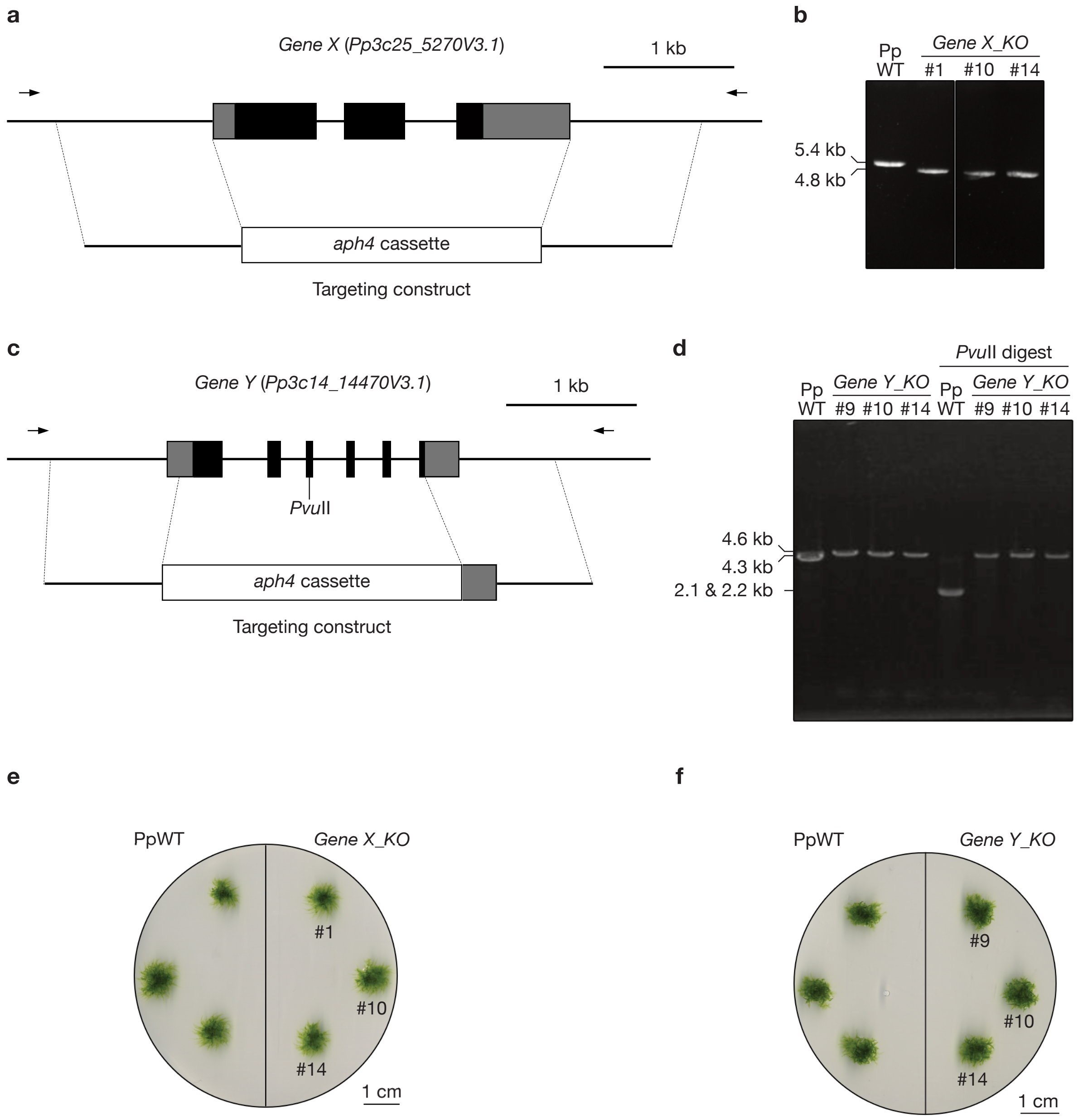
a
Gene X (Pp3c25_5270V3.1)
1 kb
aph4 cassette
Targeting construct
b
Pp WT
Gene X_KO
#1
#10
#14
5.4 kb
4.8 kb
c
Gene Y (Pp3c14_14470V3.1)
1 kb
PvuII
aph4 cassette
Targeting construct
d
PvuII digest
Pp WT
Gene Y_KO
#9
#10
#14
Pp WT
Gene Y_KO
#9
#10
#14
4.6 kb
4.3 kb
2.1 & 2.2 kb
e
PpWT
Gene X_KO
#1
#10
#14
1 cm
f
PpWT
Gene Y_KO
#9
#10
#14
1 cm

Supplementary Figure 4. Construction of *Gene X* and *Gene Y* knockout (KO) mutants

(a and c) Scheme for targeting *Gene X* and *Gene Y*. The genomic locus *Gene X* (a) or *Gene Y* (c) and the targeting construct are shown respectively in the upper and lower parts of each panel. Boxes represent exons and lines represent introns or flanking regions. Black boxes indicate the coding regions of *Gene X* or *Gene Y*, and grey boxes indicate untranslated regions. Open boxes indicate the *aph4* cassette. Arrows indicate primers used for PCR analysis in b and d. (b and d) PCR analysis of the targeted *Gene X* and *Gene Y* loci. The PCR products of the *Gene Y* locus were digested by *Pvu*II (d). (e and f) Growth of Physcomitrella wild-type (PpWT) and mutants. Plants were cultured on agar media for two weeks. Scale bars = 1 cm.

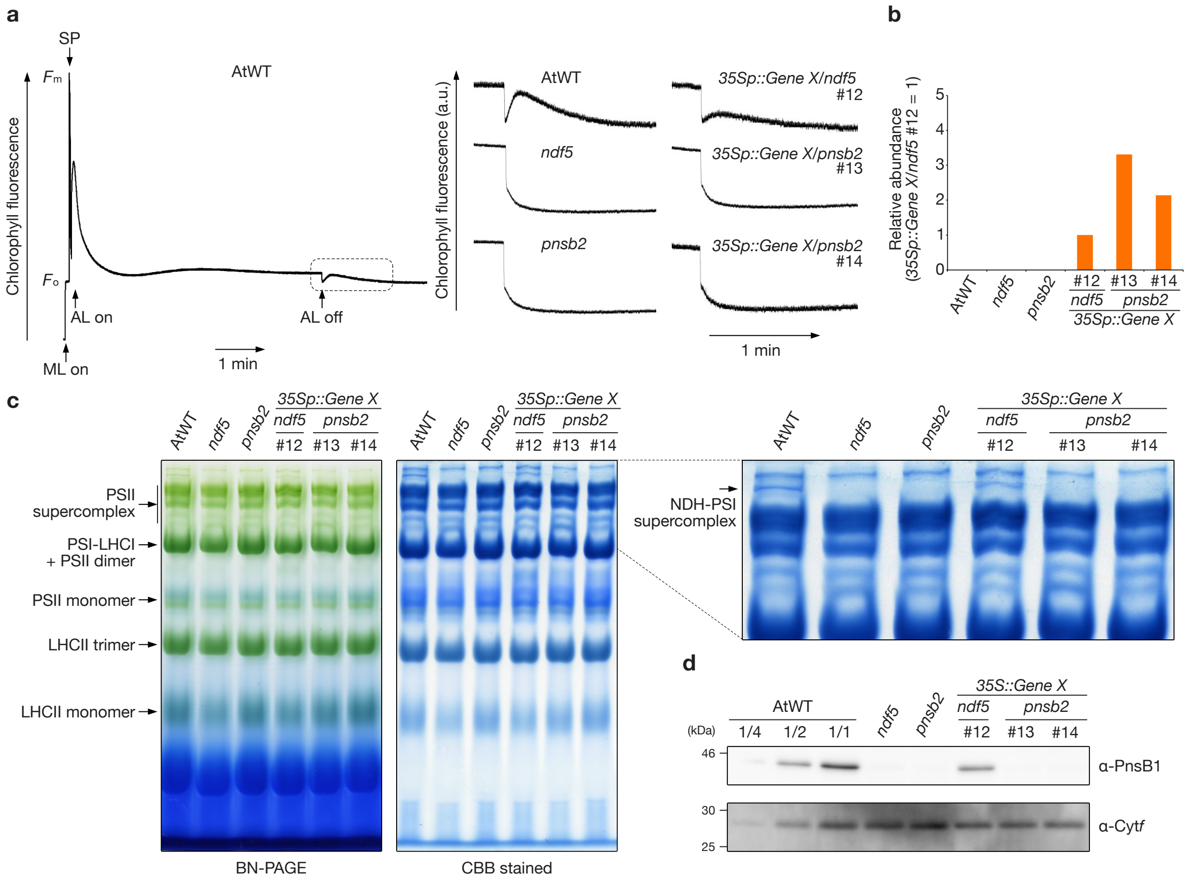

Supplementary Figure 5. *Gene X* in Physcomitrella can complement the Arabidopsis *ndf5* mutant

The same analysis as in Figure 6 was performed using independent lines. Each experiment was performed in once.

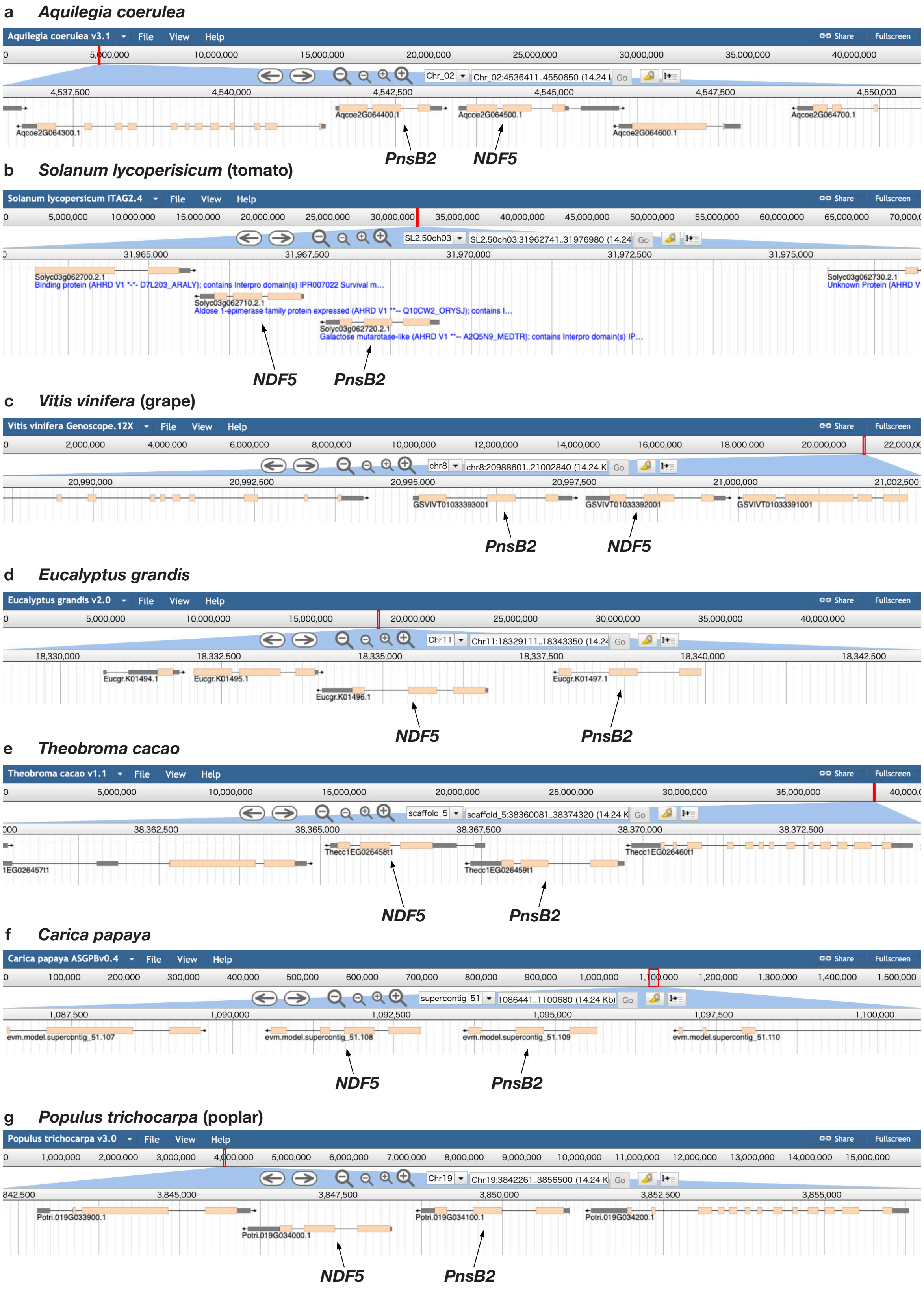

a Aquilegia coerulea
Aquilegia coerulea v3.1
Aqcoe2G064300.1
Aqcoe2G064400.1
Aqcoe2G064500.1
Aqcoe2G064600.1
Aqcoe2G064700.1
PnsB2
NDF5
b Solanum lycoperisicum (tomato)
Solanum lycopersicum ITAG2.4
Solyc03g062700.2.1
Solyc03g062710.2.1
Solyc03g062720.2.1
Solyc03g062730.2.1
NDF5
PnsB2
c Vitis vinifera (grape)
Vitis vinifera Genoscope.12X
GSVIVT01033393001
GSVIVT01033392001
GSVIVT01033391001
PnsB2
NDF5
d Eucalyptus grandis
Eucalyptus grandis v2.0
Eucgr.K01494.1
Eucgr.K01495.1
Eucgr.K01496.1
Eucgr.K01497.1
NDF5
PnsB2
e Theobroma cacao
Theobroma cacao v1.1
Thecc1EG026458t1
Thecc1EG026459t1
Thecc1EG026460t1
NDF5
PnsB2
f Carica papaya
Carica papaya ASGPBv0.4
evm.model.supercontig_51.107
evm.model.supercontig_51.108
evm.model.supercontig_51.109
evm.model.supercontig_51.110
NDF5
PnsB2
g Populus trichocarpa (poplar)
Populus trichocarpa v3.0
Potri.019G033900.1
Potri.019G034000.1
Potri.019G034100.1
Potri.019G034200.1
NDF5
PnsB2

Supplementary Figure 6. *NDF5* and *PnsB2* were tandemly arranged in some angiosperm genomes

Genome browser snapshots show the location of *NDF5* and *PnsB2* genes in some genomes of angiosperms.

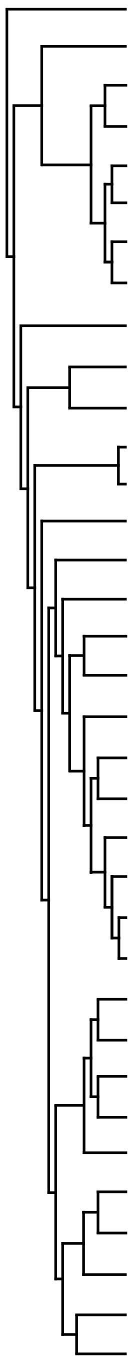

	Accession number of *PnsB2*	Accession number of *NDF5*
Amborella trichopoda	evm_27.model.AmTr_v1.0_scaffold00004.64	evm_27.model.AmTr_v1.0_scaffold00004.63
Zostera marina	Zosma39g00680	Zosma166g00100
Brachypodium distachyon	Bradi1g74570	Bradi1g08680
Oryza sativa	LOC_Os03g06230	LOC_Os03g53710
Setaria italica	Seita.9G534200	Seita.9G092700
Panicum hallii	Pahal.I00476	Pahal.I01741
Zea mays	GRMZM2G034243	GRMZM2G011373
Sorghum bicolor	Sobic.001G498300	Sobic.001G090200
Aquilegia coerulea	Aqcoe2G064400	Aqcoe2G064500
Solanum lycopersicum	Solyc03g062720.2	Solyc03g062710.2
Mimulus guttatus	Migut.J01892	Migut.J01893
Kalanchoe fedtschenkoi	Kaladp0094s0098	Kaladp1048s0001
Kalanchoe laxiflora	Kalax.0505s0012	Kalax.0381s0012
Vitis vinifera	GSVIVT01033393001	GSVIVT01033392001
Eucalyptus grandis	Eucgr.K01497	Eucgr.K01496
Citrus clementina	Ciclev10020997m	Ciclev10020539m
Theobroma cacao	Thecc1EG026459	Thecc1EG026458
Gossypium raimondii	Gorai.007G288700	Gorai.007G288600
Carica papaya	evm.model.supercontig_51.109	evm.model.supercontig_51.108
Eutrema salsugineum	Thhalv10018808m	Thhalv10011597m
Brassica rapa	Brara.F03840	Brara.F00034
Boechera stricta	Bostr.15374s0003	Bostr.6758s0034
Capsella rubella	Carubv10020412m	Carubv10009573m
Arabidopsis lyrata	AL2G19130	AL1G63430
Arabidopsis thaliana	AT1G64770	AT1G55370
Populus trichocarpa	Potri.019G034100	Potri.019G034000
Salix purpurea	SapurV1A.0308s0120	SapurV1A.0308s0130
Ricinus communis	29950.t000026	29950.t000027
Manihot esculenta	Manes.09G141900	Manes.09G142000
Linum usitatissimum	Lus10010671	Lus10020267
Glycine max	Glyma.16G041700	Glyma.16G153000
Phaseolus vulgaris	Phvul.001G110100	Phvul.004G079500
Trifolium pratense	Tp57577_TGAC_v2_gene5890	Tp57577_TGAC_v2_gene34627
Prunus persica	Prupe.3G114100	Prupe.7G003600
Cucumis sativus	Cucsa.340540	Cucsa.024050

Supplementary Figure 7. Accession numbers of *NDF5* and *PnsB2* in angiosperms

Species in which *NDF5* and *PnsB2* genes are tandemly arranged are indicated in red. The tree is based on APG IV [3]. Branch lengths do not represent phylogenetic distances.

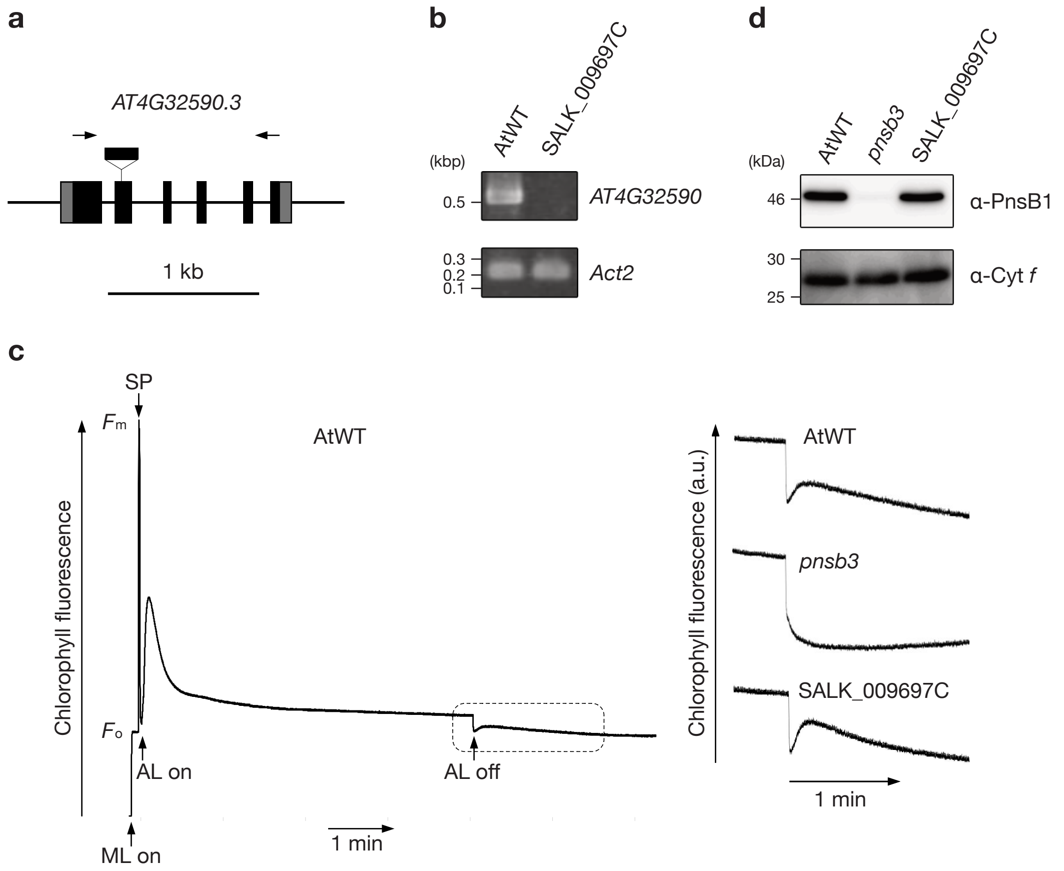

Supplemental Figure 8. ***AT4G32590*** **was not required for NDH activity.**

(a) Structure of *AT4G32590*. A position of the T-DNA insertion in the *SALK_009697C* line is indicated. Black and grey boxes indicate exons and untranslated regions, respectively. Positions of primers used for RT-PCR in B are indicated. (b) RT-PCR analysis of *AT4G32590* and *Act2* transcripts. These transcripts were amplified using cDNA from Arabidopsis wild-type (AtWT) and the *SALK_009697C* line. *Act2* was detected as a control. (c) Transient increases in chlorophyll fluorescent was monitored after turning off AL in AtWT, *psnb3*, and the *SALK_009697C* line, as in Figure 5b. A typical trace of chlorophyll fluorescent in the AtWT and the transient increases in chlorophyll fluorescent (boxed area) are shown at left and right, respectively. (d) Immunoblot analysis of chloroplast membrane proteins isolated from AtWT, *pnsb3*, and the *SALK_009697C* line. Sample loading was based on chlorophyll content. Cyt*f* was detected as a loading control. Each experiment was performed in at least twice.

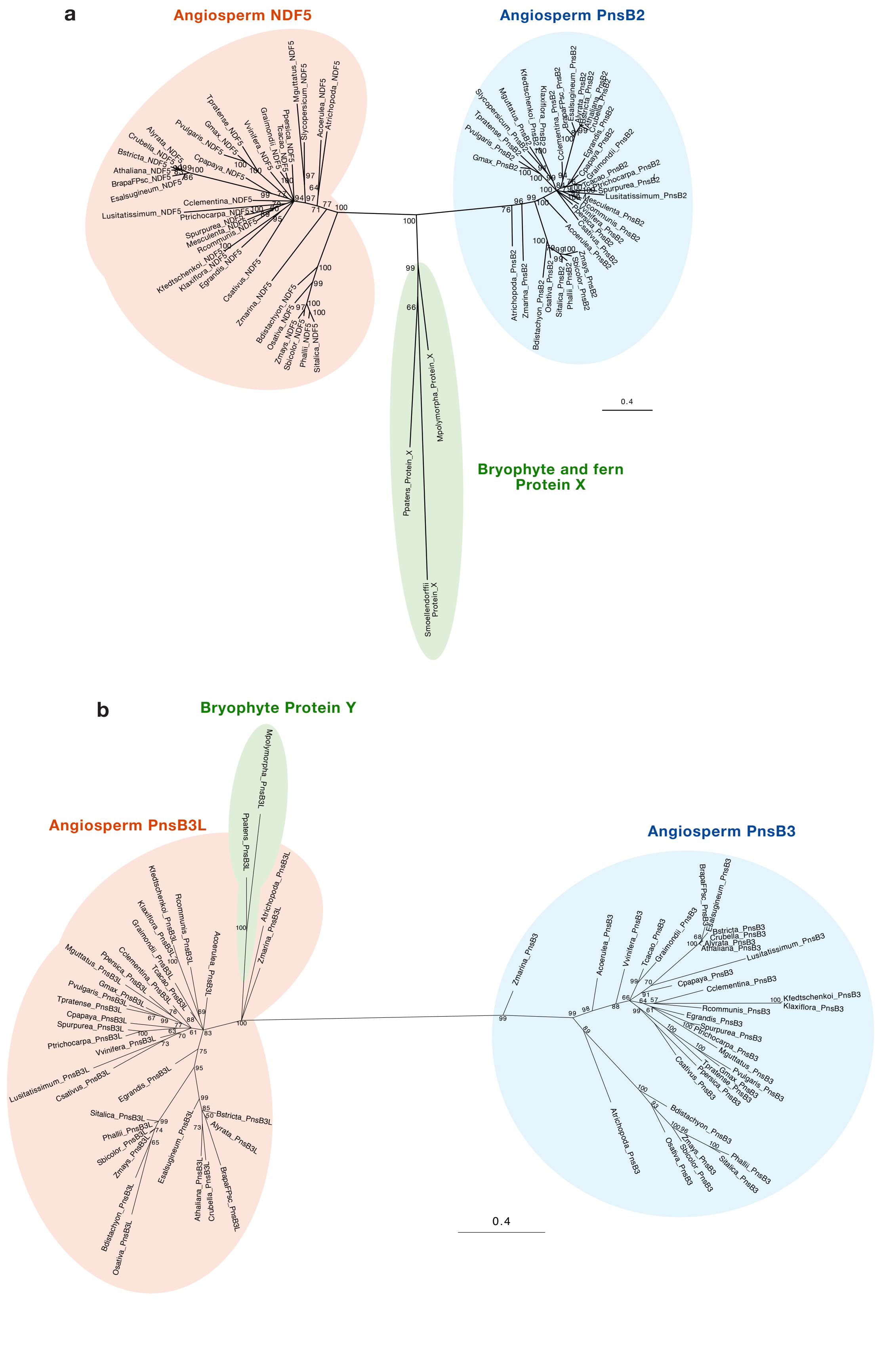
a
Angiosperm NDF5
Angiosperm PnsB2
Bryophyte and fern
Protein X
0.4
b
Bryophyte Protein Y
Angiosperm PnsB3L
Angiosperm PnsB3
0.4

Supplementary Figure 9. Phylogenetic relationships of PnsB2 and PnsB3 with their related proteins

Phylogenetic trees of (a) PnsB2, NDF5, and Protein X and (b) PnsB3, PnsB3-like (PnsB3L), and Protein Y were constructed based on Bayesian inference. Posterior probabilities for Bayesian inference are indicated. Branch length represents the estimated rate of amino acid substitution. Atrichopoda, *Amborella trichopoda*; Zmarina, *Zostera marina*; Bdistachyon, *Brachypodium distachyon*; Osativa, *Oryza sativa*; Sitalica, *Setaria italica*; Phallii, *Panicum hallii*; Zmays, *Zea mays*; Sbicolor, *Sorghum bicolor*; Acoerulea, *Aquilegia coerulea*; Slycopersicum, *Solanum lycopersicum*; Mguttatus, *Mimulus guttatus*; Kfedtschenkoi, *Kalanchoe fedtschenkoi*; Klaxiflora, *Kalanchoe laxiflora*; Vvinifera, *Vitis vinifera*; Egrandis, *Eucalyptus grandis*; Cclementina, *Citrus clementina*; Tcacao, *Theobroma cacao*; Graimondii, *Gossypium raimondii*; Cpapaya, *Carica papaya*; Esalsugineum, *Eutrema salsugineum*; Brapa, *Brassica rapa*; Bstricta, *Boechera stricta*; Crubella, *Capsella rubella*; Alyrata, *Arabidopsis lyrata*; Athaliana, *Arabidopsis thaliana*; Ptrichocarpa, *Populus trichocarpa*; Spurpurea, *Salix purpurea*; Rcommunis, *Ricinus communis*; Mesculenta, *Manihot esculenta*; Lusitatissimum, *Linum usitatissimum*; Gmax, *Glycine max*; Pvulgaris, *Phaseolus vulgaris*; Tpratense, *Trifolium pratense*; Ppersica, *Prunus persica*; Csativus, *Cucumis sativus*; Ppatens, *Physcomitrella patens*; Mpolymorpha, *Marchantia polymorpha*; Smoellendorffii, *Selaginella moellendorffii* (the accession number of Selaginella Protein X is 423216)

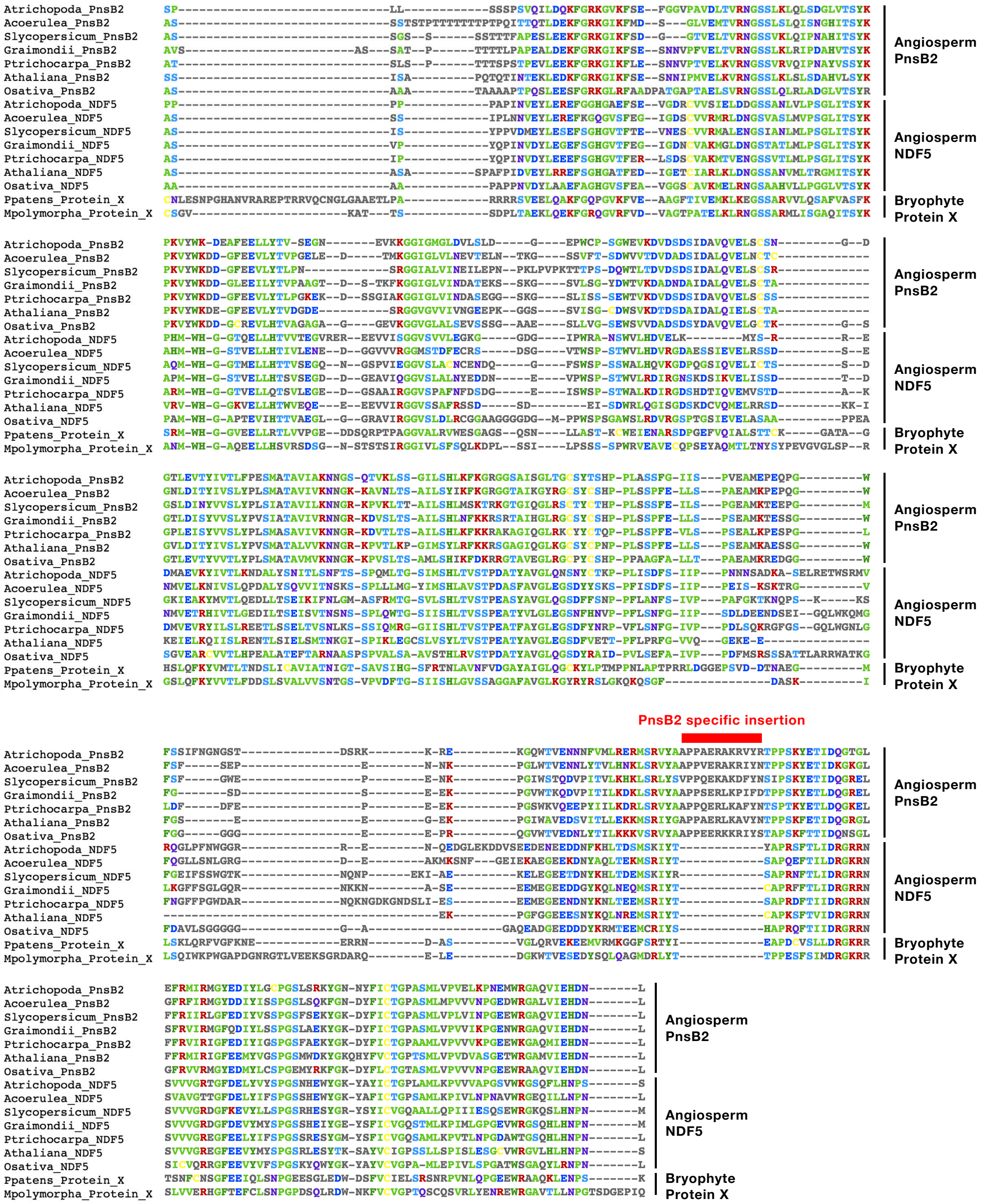

Atrichopoda_PnsB2
Acoerulea_PnsB2
Slycopersicum_PnsB2
Graimondii_PnsB2
Ptrichocarpa_PnsB2
Athaliana_PnsB2
Osativa_PnsB2
Atrichopoda_NDF5
Acoerulea_NDF5
Slycopersicum_NDF5
Graimondii_NDF5
Ptrichocarpa_NDF5
Athaliana_NDF5
Osativa_NDF5
Ppatens_Protein_X
Mpolymorpha_Protein_X
Angiosperm PnsB2
Angiosperm NDF5
Bryophyte Protein X
PnsB2 specific insertion

Supplementary Figure 10. PnsB2 contains a unique insertion at its C-terminal region. The amino acid alignment of PnsB2, NDF5, and Protein X used in the phylogenetic tree construction in Figure 4b was visualized using MView [4]. Atrichopoda, *Amborella trichopoda*; Acoerulea, *Aquilegia coerulea*; Slycopersicum, *Solanum lycopersicum*; Graimondii, *Gossypium raimondii*; Ptrichocarpa, *Populus trichocarpa*; Athaliana, *Arabidopsis thaliana*; Osativa, *Oryza sativa*; Ppatens, *Physcomitrella patens*; Mpolymorpha, *Marchantia polymorpha*

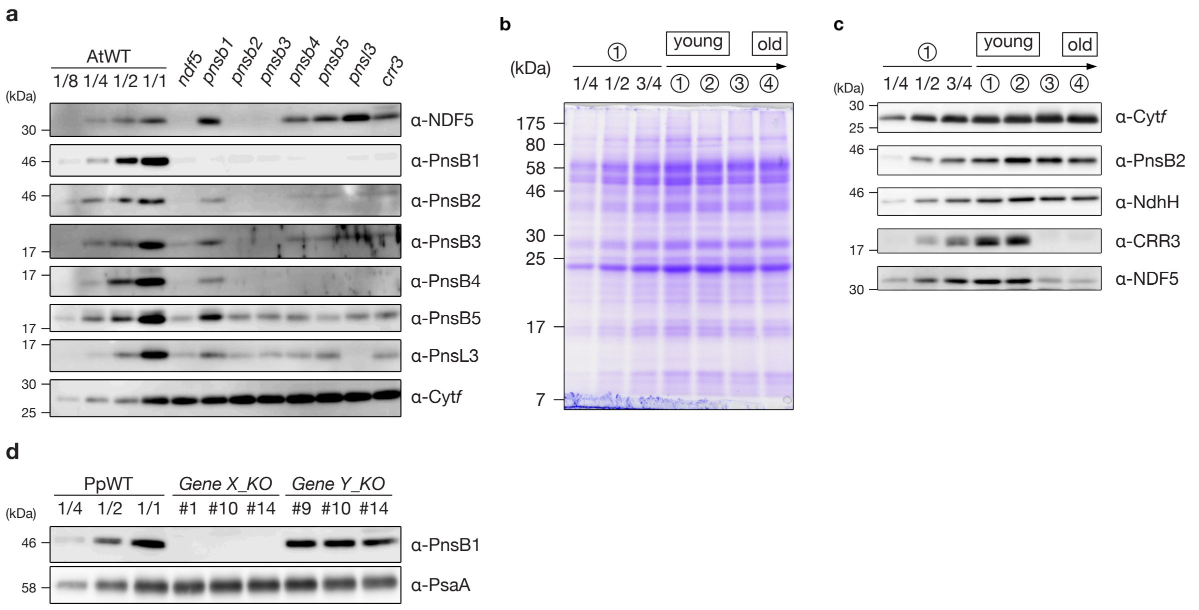

Supplementary Figure 11. Independent results in Figures 1, 3, 5, and 8

(a–c) The same analyses as in Figures 1, 3b, and 3c were performed in (a), (b), and (c), respectively. (d) The immunoblot analysis in Figures 5c and 8c was repeated using independent transgenic plants, along with a dilution series of Physcomitrella wild-type (PpWT).

Supplementary Table 1. Reciprocal Blastp results of SubB subunits in Arabidopsis and Marchantia

First, a Blastp search was performed in the Marchantia proteome using the amino acid sequences of the SubB subunits in Arabidopsis as queries. Hit sequences in Marchantia were then returned to the Blastp-search in Arabidopsis. Every sequence was used as a query after removing its putative chloroplast transit peptides predicted by ChloroP 1.1. The threshold of E-value was 1.00E^{-10}.

			1st Blastp in *M. polymorpha*				2nd Blastp in *A. thaliana*			
**Protein**	**AGI code**		**Marchantia identifier**	**Score**	**E-value**		**AGI code**	**Score**	**E-value**	**Protein**
PnsB1	AT1G15980	->	Mapoly0114s0017	378.6	9.80E-127	->	AT1G15980	378.3	5.20E-126	PnsB1
PnsB2	AT1G64770	->	Mapoly0135s0022	108.2	1.80E-26	->	AT1G55370	133.3	5.60E-35	NDF5
							AT1G64770	110.5	3.80E-27	PnsB2
PnsB3	AT3G16250	->	Mapoly0075s0083	78.2	3.40E-18	->	AT4G32590	136.7	1.20E-40	PnsB3-like
							AT3G16250	104.8	5.40E-28	PnsB3
PnsB4	AT1G18730	->	Mapoly0149s0008	81.6	2.30E-19	->	AT1G18730	87.4	2.40E-21	PnsB4
PnsB5	AT5G43750	->	Mapoly0063s0063	67	3.20E-14	->	AT5G43750	69.7	8.10E-15	PnsB5
PnsL3	AT3G01440	->	no hit							

Supplementary Table 2. Reciprocal Blastp results of SubB subunits in Arabidopsis and Physcomitrella

Reciprocal Blastp analysis was performed between Arabidopsis and Physcomitrella, as in Supplementary Table 1.

			**1st Blastp in *P. patens***				**2nd Blastp in *A. thaliana***			
**Protein**	**AGI code**		**Physcomitrella identifier**	**Score**	**E-value**		**AGI code**	**Score**	**E-value**	**Protein**
PnsB1	AT1G15980	->	Pp3c9_19250V3	398.3	6.20E-134	->	AT1G15980	398.3	1.70E-134	PnsB1
			Pp3c15_23280V3	391.3	3.30E-131	->	AT1G15980	390.2	2.10E-131	PnsB1
PnsB2	AT1G64770	->	Pp3c25_5270V3	62.4	2.00E-10	->	AT1G55370	108.2	2.40E-26	NDF5
							AT1G64770	93.6	2.40E-21	PnsB2
PnsB3	AT3G16250	->	Pp3c14_14470V3	88.2	2.70E-21	->	AT4G32590	113.2	1.10E-31	PnsB3-like
							AT3G16250	77	9.30E-18	PnsB3
PnsB4	AT1G18730	->	Pp3c16_3930V3	79	9.00E-18	->	AT1G18730	89.4	2.30E-22	PnsB4
PnsB5	AT5G43750	->	Pp3c21_10470V3	93.2	4.90E-23	->	AT5G43750	97.1	4.90E-25	PnsB5
PnsL3	AT3G01440	->	Pp3c5_4110V3	58.5	3.00E-10	->	AT4G05180	183.7	1.90E-57	PsbQ-2
							AT4G21280	181.4	1.30E-56	PsbQ-1
							AT1G14150	58.9	4.10E-11	PnsL2
							AT3G01440	57.4	2.10E-10	PnsL3
							AT2G01918	57	2.20E-10	PsbQ-like 3

Supplementary Table 3. Blastp results of NDF5, PnsB3-like, and PsbQ-2 of Arabidosis in Marchantia and Physcomitrella

Blastp search was performed in Marchantia and Physcomitrella using the amino acid NDF5, PnsB3-like, and PsbQ-2 in Arabidopsis as queries, as in Supplementary Table 1.

**Blastp in *M. polymorpha***				***A. thaliana***			**Blastp in *P. patens***		
**Marchantia identifier**	**Score**	**E-value**		**Protein**	**AGI code**		**Physcomitrella identifier**	**Score**	**E-value**
Mapoly0135s0022	120.6	6.60E-31	<-	NDF5	AT1G55370	->	Pp3c25_5270V3	79.3	1.80E-16
Mapoly0075s0083	120.2	5.00E-34	<-	PnsB3-like	AT4G32590	->	Pp3c14_14470V3	123.3	9.50E-35
				PsbQ-2	AT4G05180	->	Pp3c5_4110V3	190.3	1.60E-59
							Pp3c3_2620V3	157.1	8.00E-47
							Pp3c13_6570V3	142.1	5.00E-41
							Pp3c6_20270V3	110.5	5.40E-29
							Pp3c6_20260V3	110.5	5.40E-29

Supplementary Table 4. Primers used in this study

Primer name	Sequence (5’>3’)	Experiment
AseI_AtNDF5_antigen_F	GAATCCATTAATGGCTTCAGCTTCAGCTTCACCG	Construction for recombinant Arabidopsis NDF5 protein expression
XhoI_AtNDF5_antigen_R	CCGCTCGAGGGAATTAGGATTGTGAAGATGTAGGA	
D-TOPO_inf_AtNDF5_5UTR_F	AGCAGGCTCCGCGGCCGCCAGATTGTGAGTGTTGTCTTCTTC	Construction for Arabidopsis transformation
Gene_X_AtNDF5_5UTR_R	GATCCCCACCATGTCGCACATTGTTACGGCTCTGAAAACGTTTCA	
Gene_X_ATG_F	ATGTGCGACATGGTGGGGATCCACG	
(D-TOPO_inf_) Gene_X_stop_R	AAGCTGGGTCGGCGCGCCCTCACTTACTTGGGTTCTCCAG	
Gene_X_stop_AtHSP18.2_term_F	CTGGAGAACCCAAGTAAGTGAATATGAAGATGAAGATGAAA	
D-TOPO_inf_AtHSP18.2_term_R	AAGCTGGGTCGGCGCGCCCCTTATCTTTAATCATATTCC	
Gene_X_qRT_F	GCAGGTAGCCAGTCGAATTT	RT-qPCR in Arabidopsis
Gene_X_qRT_R	CAAGTGGTCGATAAGGCAATCT	
AtEF1α_qRT_F	AAACTTAAGGCCGAGCGTGA	
AtEF1α_qRT_R	CCTGGGGCATCAATGACTGT	
EcoRV_inf_XbaI_Upstream_Gene_X_F	atcgataagcttgattctagaTGAATCAAGTAAAAAAATCGTAA	Construction for *Gene X* knockout in Physcomitrella
EcoRV_inf_Upstream_Gene_X_R	cgaagttatttcgatAGACGCTGAACGAGTACCGAAAAC	
XmaI_inf_downstream_Gene_X_F	ggatcgcatgcccggAGTTAAGCTTGGTAAACGTTTGAA	
XmaI_inf_downstream_Gene_X_R	tatgtctagacccggAGATAAAATTCCACATCGGTTGTA	
EcoRV_inf_Upstream_Gene_Y_F	atcgataagcttgatCCGCTTGTGACATCTTGCTCTTTAGG	Construction for *Gene Y* knockout in Physcomitrella
EcoRV_inf_Upstream_Gene_Y_R	cgaagttatttcgatGGGCTTGGATGCTTCCTGTCAGCA	
XmaI_inf_downstream_Gene_Y_F	ggatcgcatgcccggATTTCTAGAGGAGGTGATGACGTCAAG	
XmaI_inf_downstream_Gene_Y_R	tatgtctagacccgggTTAAGTCATTATGTTAAAAAGTCA	
Gene_X_genotype_F	TGCAACATGACATTTGTGGATTTGG	Genotyping PCR of *Gene X* knockout in Physcomitrella
Gene_X_genotype_R	TCATACGACCACCATTACCATCACC	

Supplementary Table 4. Continued

Primer name	Sequence (5’>3’)	Experiment
Gene_Y_genotype_F	TTGCAGCATACGAGAATCCGTTGG	Genotyping PCR of *Gene Y* knockout in Physcomitrella
Gene_Y_genotype_R	CAATCTCCTCCTATTCATTGTCCCAAG	
Gene_X_RT_F	ATGAAGCTGAAAGAAGGTTCGAGTGC	RT-PCR in Physcomitrella
Gene_X_RT_R	TCACTTACTTGGGTTCTCCAGTTTCTG	
Gene_Y_RT_F	ATGGACACGCTATTGCATTCGCG	
Gene_Y_RT_R	TCACTTCTTCTTCTGGGGAAGTC	
PpPnsB4_RT_F	ATGGCTATGGCGCAAGCAGTTCTG	
PpPnsB4_RT_R	ACTTGTGAGCTCTGCAAATTGTTGAGAG	
AT4G32590_RT_F	ATGGCTTCGTTAGGTTTCAACCTCG	RT-PCR in Arabidopsis
AT4G32590_RT_R	TTAATTCACTTCTTCCACTGTGGA	
AtAct2_RT_F	ACACTGTGCCAATCTACGAGGGTT	
AtAct2_RT_R	ACAATTTCCCGCTCTGCTGTTGTG	

Supplementary Table 5. Accession numbers of PnsB3 and PnsB3-like orthologs in angiosperms

Species	Accession number of PnsB3	Accession number of PnsB3-like
Aquilegia coerulea	Aqcoe7G162000.1.p	Aqcoe7G196800.1.p
Arabidopsis lyrata	AL3G28950.t1	AL7G19180.t1
Arabidopsis thaliana	AT3G16250.1	AT4G32590.3
Amborella trichopoda	evm_27.model.AmTr_v1.0_scaffold00109.159	evm_27.model.AmTr_v1.0_scaffold00118.15
Brachypodium distachyon	Bradi1g27280.2.p	Bradi2g17200.3.p
Brassica rapa	Brara.A03067.1.p	Brara.A00536.1.p
Boechera stricta	Bostr.28625s0065.1.p	Bostr.7867s1109.1.p
Citrus clementina	Ciclev10026544m	Ciclev10009693m
Carica papaya	evm.model.supercontig_48.142	evm.model.supercontig_91.15
Capsella rubella	Carubv10014651m	Carubv10005956m
Cucumis sativus	Cucsa.359350.1	Cucsa.162010.2
Eucalyptus grandis	Eucgr.F03055.2.p	Eucgr.L02133.1.p
Eutrema salsugineum	Thhalv10021523m	Thhalv10026415m
Glycine max	Glyma.15G018000.1.p	Glyma.17G205000.1.p
Gossypium raimondii	Gorai.011G072900.1	Gorai.013G238500.1
Kalanchoe fedtschenkoi	Kaladp0011s0475.1.p	Kaladp0032s0242.1.p
Kalanchoe laxiflora	Kalax.0371s0028.1.p	Kalax.0027s0038.1.p
Linum usitatissimum	Lus10038292	Lus10041038
Mimulus guttatus	Migut.I00927.1.p	Migut.E01491.1.p
Oryza sativa	LOC_Os07g30670.1	LOC_Os05g48160.1
Panicum hallii	Pahal.B03952.1	Pahal.C02020.1
Prunus persica	Prupe.3G063600.1.p	Prupe.1G467300.1.p
Populus trichocarpa	Potri.001G186800.1	Potri.006G247300.2
Phaseolus vulgaris	Phvul.005G170200.1.p	Phvul.001G055300.1.p
Ricinus communis	30146.m003541	29794.m003498
Sorghum bicolor	Sobic.002G305700.1.p	Sobic.009G225600.1.p
Setaria italica	Seita.2G318000.1.p	Seita.3G153500.1.p
Salix purpurea	SapurV1A.2712s0010.1.p	SapurV1A.0043s0130.1.p
Theobroma cacao	Thecc1EG028838t1	Thecc1EG038311t1
Trifolium pratense	Tp57577_TGAC_v2_mRNA38477	Tp57577_TGAC_v2_mRNA20797
Vitis vinifera	GSVIVT01017041001	GSVIVT01035716001
Zostera marina	Zosma5g02060.1	Zosma74g01180.1
Zea mays	GRMZM2G032253_P01	GRMZM2G481261_P01

Supplementary References

1. Sakamoto, W. *et al.* Coordinated regulation and complex formation of YELLOW VARIEGATED1 and YELLOW VARIEGATED2 , chloroplastic FtsH metalloproteases involved in the repair cycle of photosystem II in Arabidopsis thylakoid membranes. *Plant Cell* **15**, 2843–2855 (2003).
2. Zhang, D. *et al.* The FtsH protease heterocomplex in *Arabidopsis*: dispensability of type-B protease activity for proper chloroplast development. *Plant Cell* **22**, 3710–3725 (2010).
3. Chase, M. W. *et al.* An update of the Angiosperm Phylogeny Group classification for the orders and families of flowering plants: APG IV. *Bot. J. Linn. Soc.* **181**, 1–20 (2016).
4. Brown, N. P., Leroy, C. & Sander, C. MView: a web-compatible database search or multiple alignment viewer. *Bioinformatics* **14**, 380–381 (1998).
